# Integrated cold resistance subgrade system utilizing oil shale waste and XPS insulation for sustainable infrastructure in seasonal frozen regions

**DOI:** 10.1038/s41598-025-17133-8

**Published:** 2025-08-27

**Authors:** Leilei Han, Yiqiang Gao

**Affiliations:** 1 Postdoctoral Workstation of Shaanxi Provincial Transport Planning Design and Research Institute Co., Ltd., Xi’an, 710065 China; 2 School of transportation, Jilin University, Changchun, 130022 China; 3 Xi ’an Chuangmiao Technology Co., Ltd., Xi’an, 710065 China

**Keywords:** Oil shale waste, Cold resistance structure, Extruded polystyrene, In-situ test, Dynamic stress response, Environmental sciences, Engineering, Materials science

## Abstract

To address the environmental concerns of oil shale waste (OSW) accumulation and improve road engineering sustainability, this paper proposes a novel cold resistance structure (CRS) incorporating extruded polystyrene (XPS) insulation plates and OSW-modified soil. OSW primarily consists of two components: residual semi-coke from retorting processes and combustion-derived ash residues. The improper disposal of accumulated OSW poses significant environmental risks. Following a comprehensive feasibility assessment, this study identifies the application of OSW in highway subgrade construction as an eco-friendly solution that achieves triple objectives - waste valorization, pollution mitigation, and alleviation of material shortages in road infrastructure. Targeting the freeze-thaw challenges prevalent in northeast China’s road structures, the CRS system combines XPS insulation technology with OSW-modified subgrade soil through three key phases. First, the optimal XPS plate thickness was determined using thermal resistance equivalence principles. Second, controlled freeze-thaw experiments employing a specialized unidirectional testing system evaluated the CRS’s frost resistance through triplicate comparative trials. Third, field validation involved constructing a CRS test road and conducting in-situ assessments of bearing capacity and dynamic stress responses, with conventional sand-gravel subgrade sections serving as controls. Environmental impacts and economic viability were systematically analyzed. Results demonstrate that the CRS system reduces the subgrade freezing depth by up to 52.8%, limits surface water migration by over 60%, and decreases dynamic stress amplitudes by more than 50% compared to conventional structures. The effective stress buffering depth of the XPS insulation is equivalent to an 89.75 cm thick gravel layer, while subgrade deflection is reduced by 21%. Additionally, the CRS system achieves a 43% reduction in material cost per kilometer and enables the reuse of over 9300 tons of solid waste, offering both economic and environmental benefits.

## Introduction

**Fig. 1:**
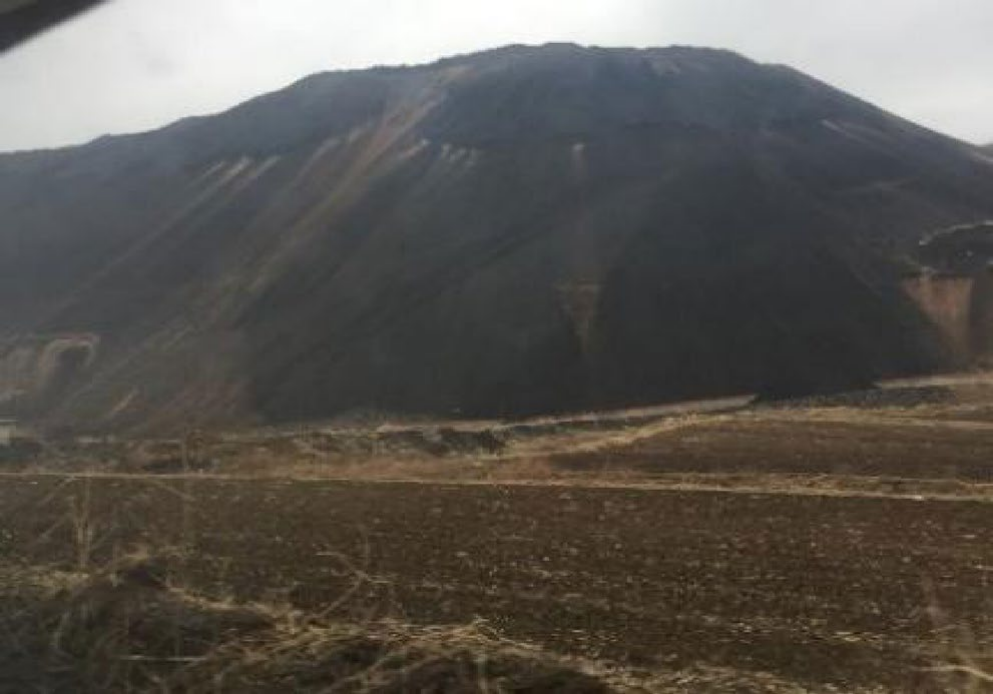
Mountain of oil shale waste residue.

Oil shale, a sedimentary rock containing organic matter, serves as a low-grade fuel for energy production. Jilin Province possesses proven reserves of 108.6 billion tons, representing over 80% of China’s total reserves^1^. Through low-temperature retorting, shale oil—a petroleum-like substance—is extracted via thermal decomposition of organic components. Subsequent hydrocracking yields various petrochemical products including gasoline, diesel, and paraffin derivatives^2^. Alternative utilization involves direct combustion for power generation, though both processes generate substantial waste: over 90% residual semi-coke from retorting and 40–50% ash from combustion^2,3^. Current disposal practices predominantly involve on-site stockpiling (Fig. 1), creating significant environmental challenges.

The expanding exploitation of oil shale resources exacerbates waste accumulation, agricultural land occupation, and ecological risks^4,5^. While OSW has been repurposed in microcrystalline glass production^6^, pigments^7^, and construction materials^8–10^, these applications inadequately address mass accumulation due to limited consumption capacities.

Concurrently, global sand shortages in road construction^11^ necessitate sustainable alternatives. OSW utilization in subgrade engineering emerges as a dual-benefit solution, simultaneously mitigating material scarcity and waste accumulation^12^. Our research team systematically evaluated OSW’s viability as subgrade filler per JTG D30-2015 specifications^13^, analyzing physicochemical properties, leaching toxicity, and pH levels. Results confirm OSW’s environmental safety for highway applications. Building on fly ash-modified soils’ demonstrated frost resistance, we developed an OSW-fly ash modified silty clay composite optimized for seasonal freeze-thaw conditions. Laboratory testing established optimal mix ratios, California Bearing Ratio (CBR), and freeze-thaw resilient modulus values, demonstrating superior mechanical performance and frost durability^14,15^.

To enhance frost damage mitigation in seasonal frozen regions, this study proposes a Cold Resistance Structure (CRS) combining extruded polystyrene (XPS) insulation with OSW-modified soil. Material characterization confirmed XPS’s stable thermal properties^12^, supporting its integration into subgrade systems. The research methodology encompasses:

1. Unidirectional freezing tests comparing thermal regulation performance.
2. Full-scale test road construction with in-situ evaluations of bearing capacity and dynamic stress response.
3. Comparative environmental and economic assessments.

Results demonstrate CRS’s effectiveness in preventing frost penetration, reducing structural load impacts, and enabling large-scale OSW recycling, while maintaining cost-effectiveness. This integrated approach presents a sustainable solution for cold region infrastructure development.

In contrast to existing cold resistance solutions, such as geosynthetic insulation materials (e.g., polyethylene foam, glass foam) and conventional waste substitution materials (e.g., fly ash, construction and demolition waste), the proposed CRS system presents multiple advantages. While geosynthetic materials often suffer from high costs, moisture susceptibility, and limited long-term mechanical performance, the extruded polystyrene (XPS) insulation used in CRS exhibits superior thermal stability, mechanical strength, and water resistance. Additionally, typical waste substitution approaches primarily focus on partial replacement of soil or aggregates, with limited enhancement to freeze-thaw resilience or structural load-bearing capacity. The CRS integrates both advanced insulation and high-performance OSW-modified filler, achieving comprehensive improvements in frost protection, mechanical strength, environmental sustainability, and cost-efficiency. This synergy of thermal and structural optimization underscores the novelty and practical value of the proposed system.

Although oil shale waste (OSW) has been preliminarily studied for use in cement, bricks, and backfilling concrete, its application in road subgrade engineering remains extremely limited. Most previous studies have focused on laboratory-scale material characterization without integrating OSW into full subgrade structures or evaluating long-term performance under freeze-thaw conditions. Furthermore, little attention has been paid to the synergistic use of OSW with thermal insulation technologies to simultaneously address frost heave damage and solid waste utilization.

This study fills that gap by developing a composite subgrade system (CRS) that combines OSW-modified soil with extruded polystyrene (XPS) insulation, and by validating the system through large-scale field tests and comprehensive environmental and economic assessments. This integrated approach not only promotes the circular reuse of industrial by-products, but also offers a novel solution for infrastructure resilience in cold regions, thereby advancing both green construction and subgrade engineering.

## Materials and methods

### Raw materials

#### Oil shale waste

The oil shale waste (OSW) used in this study was sourced from Longteng Energy Development Co., Ltd., located in the Luozigou Oil Shale Industrial Park, Wangqing County, Jilin Province. OSW consists of a mixture of oil shale ash and oil shale semi-coke, with particle sizes ranging from 0.075 mm to 150 mm. The chemical composition of OSW was analyzed using X-ray diffraction (XRD), and the results are presented in Table 1. No harmful minerals were detected.

**Table 1:** Chemical composition of OSW (%).

SiO_2_	Al_2_O_3_	Fe_2_O_3_	CaO	MgO	Na_2_O	K_2_O	TiO_2_	Loss on ignition
56.28	13.44	7.22	6.42	2.49	2.31	1.84	0.59	8.56

#### Fly Ash

Fly ash is a common industrial byproduct. Its addition to silty clay has been shown to enhance the insulation performance of the material^16^. The fly ash used in this study is derived from the combustion of pulverized coal in power plants and is classified as Level F and Class I according to the specification^17^. The chemical composition of the fly ash is presented in Table 2.

**Table 2:** Chemical composition of fly Ash (%).

SiO_2_	Al_2_O_3_	Fe_2_O_3_	CaO	MgO	K_2_O	TiO_2_	SO_3_	Loss on ignition
51.00	34.80	5.70	1.80	1.95	0.77	1.42	0.36	1.66

#### Silty clay

The silty clay taken from the borrow earth pits of the test road (Yushu-Songyuan Expressway) is uniform and free of impurities. The basic indexes of the silty clay are determined according to the specification^18^, and the results are demonstrated in Table 3.

### OSW modified soil

Fly ash-modified soil has been proven to exhibit excellent thermal insulation and freeze-thaw resistance, making it suitable for road subgrades in seasonally frozen areas^14,19^. This inspired the idea of incorporating fly ash into OSW soil. In accordance with relevant specifications^18,20^, a series of experiments were conducted to determine the optimal mix ratio of OSW, fly ash, and silty clay. These tests included compaction tests, CBR tests, resilient modulus tests, and Atterberg limits tests, all performed in strict compliance with specification requirements. Readers interested in the detailed mix design process are encouraged to refer to previous studies by our research group^21^.

Based on the fundamental requirements for subgrade filler and following the principle of maximizing OSW utilization, the optimal dry mass ratio of OSW, fly ash, and silty clay was determined to be 2:1:2. The test results for this ratio are presented in Table 3, with the parameters of silty clay included for comparison.

**Table 3:** Engineering parameters of the OSW modified soil and silty clay.

Index	Modified soil	Silty clay	Specification requirements
Optimum moisture content	12.8%	12.1%	--
Maximum dry density	1.67 g/cm^3^	1.92 g/cm^3^	--
CBR	44.9%	0.9%	≥ 8% (Highway)
Resilient modulus	98.8 MPa	51.5 MPa	≥ 70 MPa (The traffic load level is extremely heavy)
Liquid limit	39.4%	35.0%	--
Plastic limit	26.9	21.1%	--
Plasticity index	12.5	13.9	--

In terms of the CBR and resilient modulus indices specified for subgrade soil, OSW-modified soil shows significant improvement compared to silty clay. It meets the required standards and is suitable for use as highway subgrade filler.

### Structural design of the CRS

The design of XPS plates aims to maximize cold resistance while ensuring construction feasibility and meeting the mechanical requirements of the road structure. The primary mechanical consideration is to ensure that the XPS plates can withstand driving loads and structural dead weight without failure. Cold resistance is evaluated based on the thermal resistance equivalence principle, ensuring that the structure beneath the XPS plates remains unaffected by negative temperature energy.

**Fig. 2:**
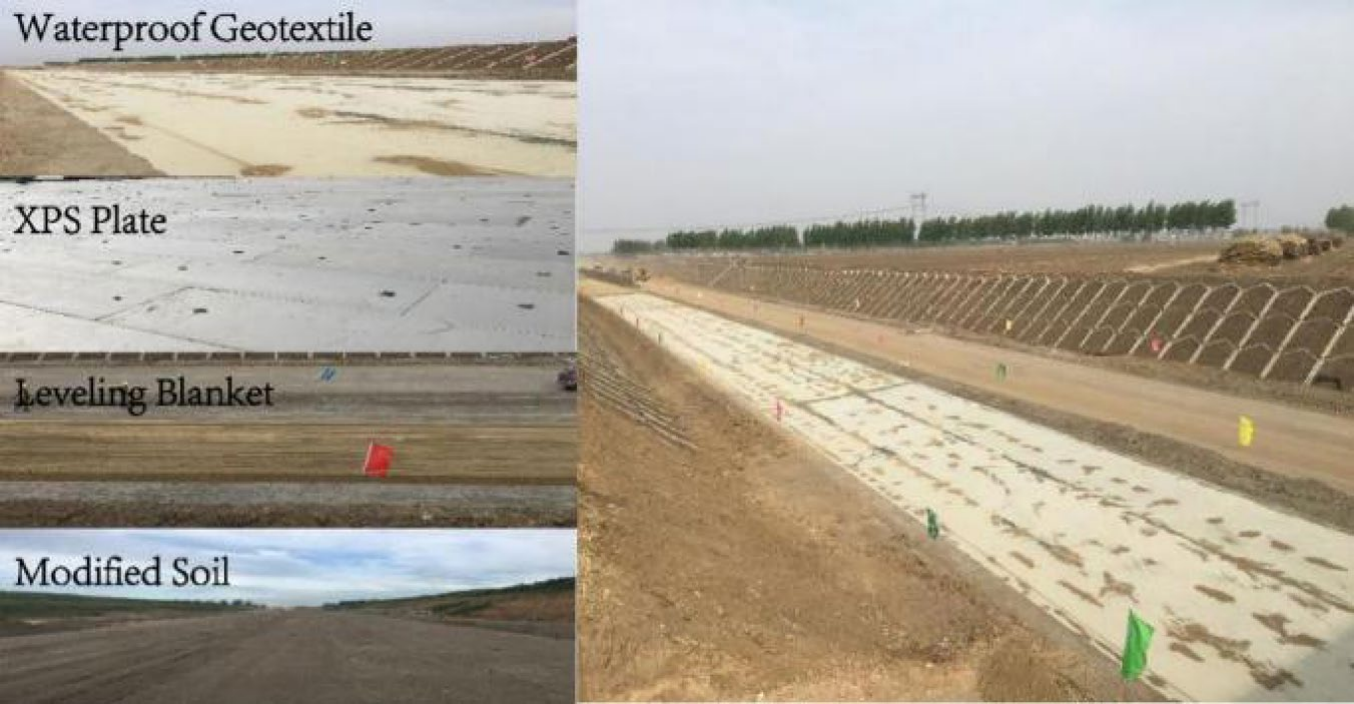
Construction structure drawing of the CRS.

The parameters of the XPS plates used in this study are listed in Table 4. Based on test road data, the CRS is designed with a 5 cm XPS plate layer and a 30 cm OSW-modified soil layer. The detailed calculation process is available in a published study^22^. A photograph of the on-site construction is shown in Fig. 2.

**Table 4:** Parameters of the XPS plates.

Apparent density	Compressive strength	Volume water absorption	Thermal conductivity(W/m.℃)	
0.04 g/cm^3^	410 kPa	0.39%	0.0245(20℃)	0.0243(−18℃)

### One-way freezing test

**Fig. 3:**
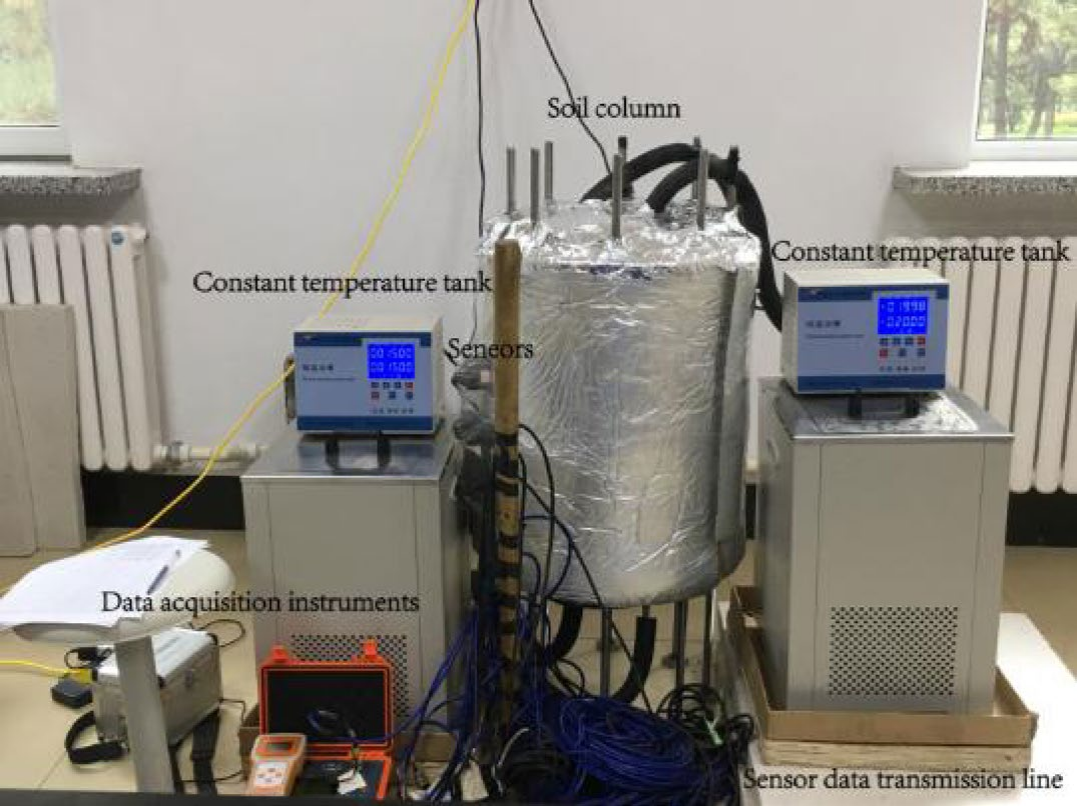
One-way freeze-thaw test system.

The one-way freeze-thaw test system used in this study enables rapid and accurate acquisition of temperature, moisture, and stress field data for different subgrade materials and structures during the freeze-thaw process. The system primarily consists of a soil column cylinder, two constant temperature tanks, sensors, and data acquisition instruments, as shown in Fig. 3.

The sensors used in this test include:

(1) Temperature Sensor: The XHS-DS18 intelligent temperature sensor, manufactured by Changsha Xianghao Electronics Co., Ltd., was used to measure temperature. It has a measuring range of −55℃ to 125℃, an accuracy of ± 0.5℃, and a resolution of 0.25℃.

(2) Moisture Sensor: The HL-TR03-RS485-H moisture sensor was employed to collect moisture content data. It has a measuring range of 0–100%, with an accuracy of ± 2% (m³/m³) within the 0–50% (m³/m³) range, allowing for precise measurement of moisture content in frozen soil.

(3) Stress Sensor: The vibrating wire stress sensor (SZZX-EA04B), produced by Changsha Sanzhi Electronic Technology Co., Ltd., was used to measure stress. It has a measuring range of 0–0.2 MPa, an accuracy of less than 0.05% F.S, and a temperature measuring range of −30℃ to 60℃.

Three groups of tests were conducted on silty clay, OSW-modified soil, and the CRS. The soil column height in all tests was 45 cm, with the CRS group consisting of a 40 cm OSW-modified soil column and a 5 cm XPS plate on the surface. To comprehensively capture the internal state of the soil column during testing, the top surface was set as the reference plane (0 cm). The sensor layout was as follows:

1. Temperature sensors were placed at depths of 5 cm, 7 cm, 10 cm, 15 cm, 20 cm, 25 cm, 30 cm, 35 cm, and 40 cm.
2. Moisture sensors were positioned at 15 cm, 25 cm, and 35 cm.
3. Stress sensors were installed at the same depths as the moisture sensors, ensuring that sensors did not come into contact with each other to prevent interference in data collection.

The test was conducted in Yushu City, Changchun. Based on local temperature data, the upper constant temperature tank was set to −20℃, while the lower constant temperature tank was maintained at 15℃. The temperature of the constant temperature tank usually reaches the set temperature in a very short time, and then the test officially starts timing. Sensor data were collected every hour until the internal temperature, moisture content, and stress data remained stable for 24 consecutive hours, at which point the test was concluded.

The upper and lower constant temperature tanks were activated simultaneously. Temperature was recorded hourly until thermal equilibrium was achieved for 24 consecutive hours. Moisture and stress data were collected at the same intervals. Data smoothing was performed using a moving average filter (window size = 3) to eliminate noise. The freezing depth was determined based on the 0 °C isotherm, and water content trends were analyzed using interpolation mapping in Origin 2023 software.

In this experiment, the primary control parameters include the upper and lower boundary temperatures (set at − 20 °C and + 15 °C, respectively), soil column height (45 cm), initial moisture content (measured as 12.8% for OSW-modified soil), and sensor layout positions. The test duration was determined based on the time required for the temperature and moisture data to stabilize over 24 consecutive hours.

The evaluation indices include:

(1) Freezing depth, defined by the depth of the 0 °C isotherm.

(2) Water migration, assessed by the change in volumetric water content at specified depths.

(3) Stress variation, reflecting internal force response due to freezing.

These indices comprehensively evaluate the thermal insulation and freeze-thaw resistance of the subgrade materials.

Experimental Procedure and Data Processing:

Prior to testing, all soil samples were compacted to optimal moisture content and maximum dry density based on standard compaction results. The soil columns were constructed in layers and sealed to prevent evaporation. Temperature, moisture, and stress sensors were embedded at pre-defined depths, ensuring uniform spatial distribution and minimal interference.

### In-situ dynamic load test

**Fig. 4:**
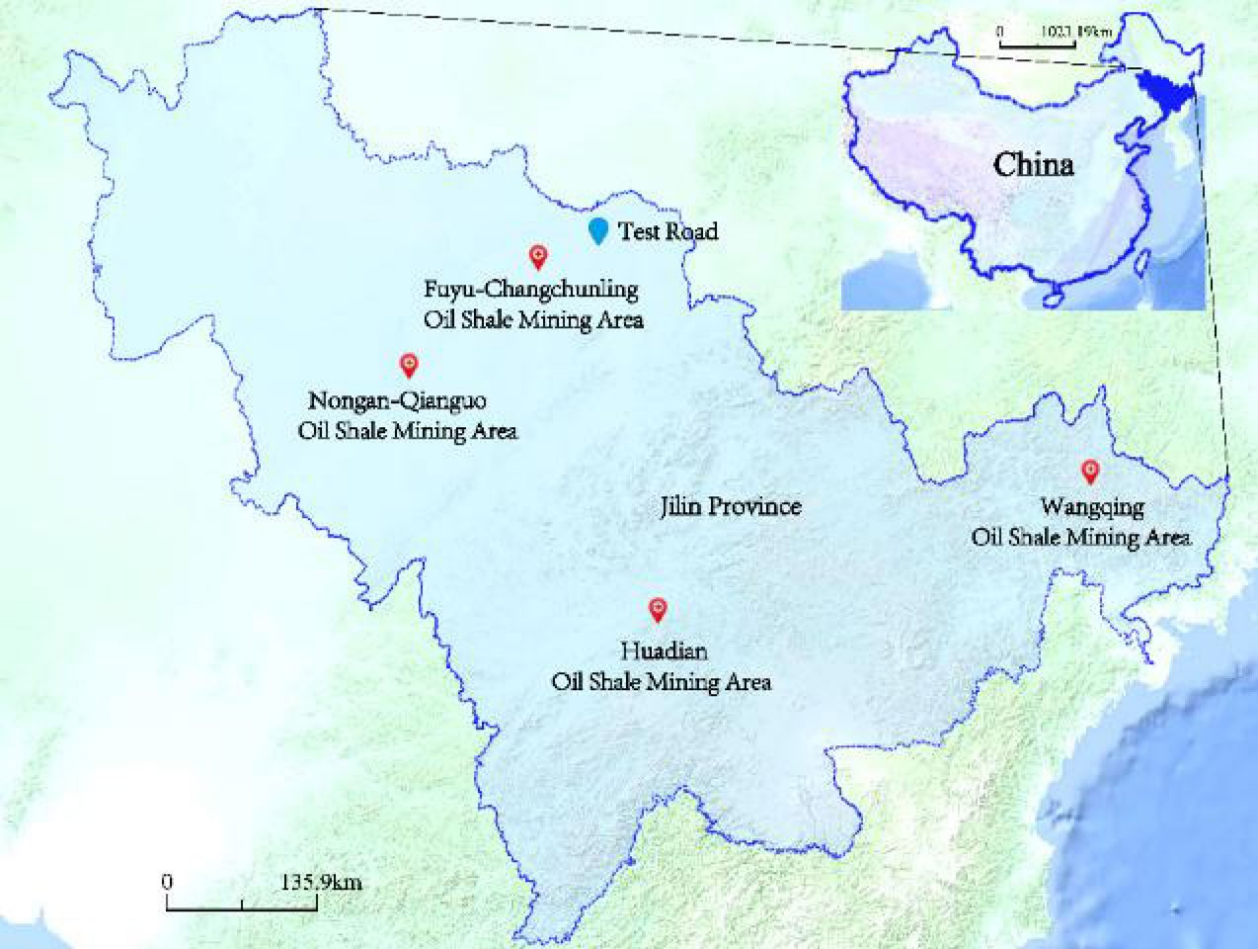
The geographical location of the test road and the distribution of adjacent oil shale mining areas. The figure was created by the author using LocaSpace Viewer version 4.0.7 (LocaSpace Viewer, http://www.tuxingis.com/locaspace.html).

The in-situ test is conducted to evaluate the road performance of the CRS, as it is the most effective method for assessing the structural behavior of a roadbed. This test provides a realistic reflection of the structural performance of the new roadbed design. The geographical location of the test road and the distribution of adjacent oil shale mining areas are shown in Fig. 4.

**Fig. 5:**
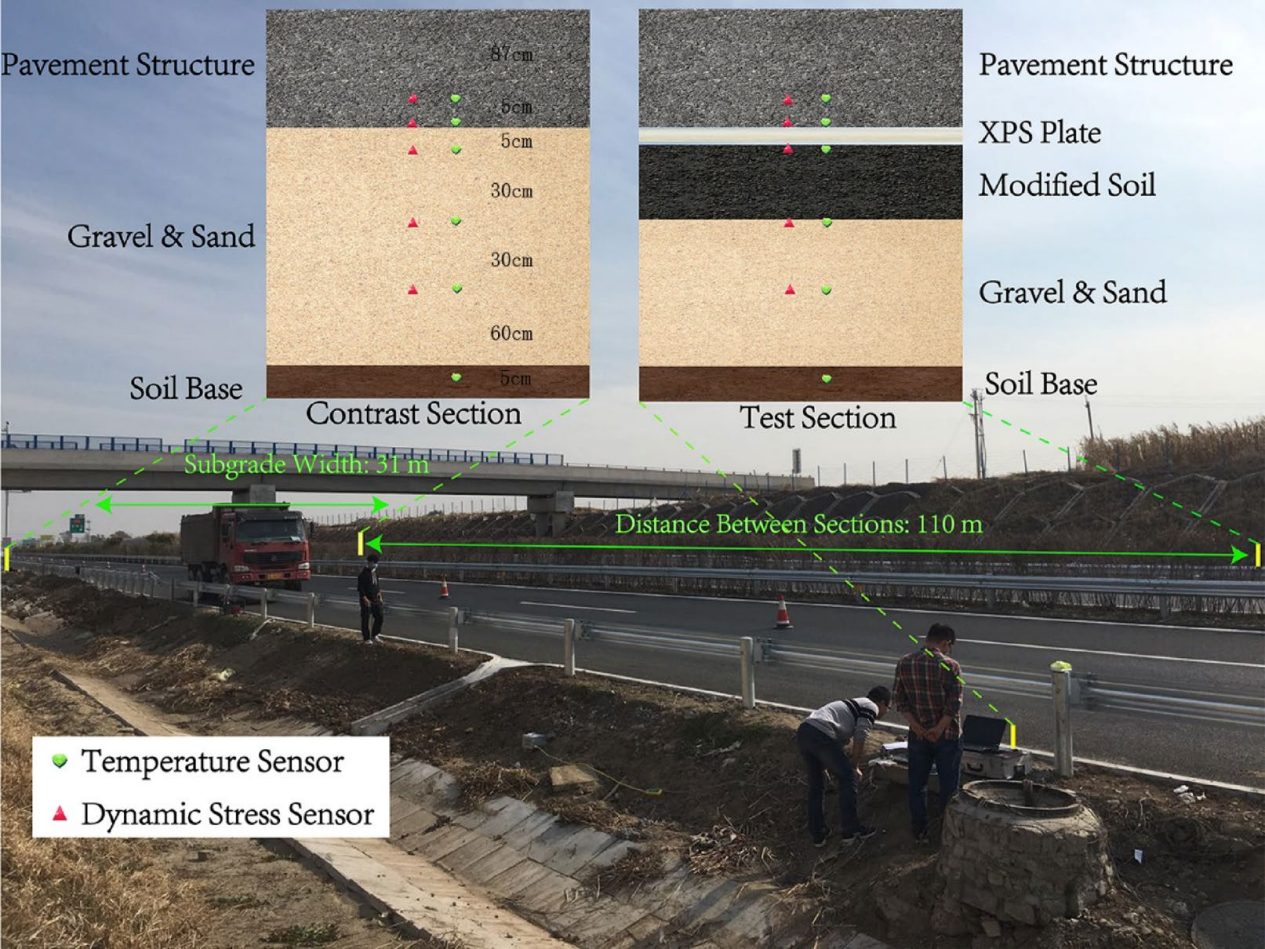
Section structure and sensor distribution map of in-situ test.

The structural design of the test road includes a pavement structure with a total thickness of 92 cm. The roadbed is in a wet condition, and conventional subgrade design typically consists of 80–120 cm of sand and gravel fill. In contrast, the test road subgrade comprises a 5 cm XPS plate layer and a 30 cm OSW-modified soil layer. However, the performance of CRS has not yet been validated through practical applications. For engineering safety considerations, only the upper 35 cm of sand and gravel in the test road subgrade was replaced with CRS, as illustrated in Fig. 5. During road construction, temperature and dynamic stress sensors were embedded at the corresponding depths in both the test and control sections.

To comprehensively analyze the test road’s response to dynamic loads, in-situ testing was conducted at different speeds and vehicle weights. The specific loading scheme is detailed in Table 5. The standard load P is 0.7 MPa, calculated based on the rear axle load and tire contact area according to the specifications^23^. Under each loading condition, vehicles passed through both the test section and control section at speeds of 5 km/h, 20 km/h, 40 km/h, 60 km/h, and 80 km/h.

**Table 5:** Loading scheme for in-situ tests.

Design Load	Front Axle Load(T)	Rear Axle Load(T)	Actual Load
0.6*P	5.92	12.23	0.63*P
0.8*P	5.91	16.24	0.84*P
P	6.90	20.01	1.03*P
1.2*P	7.16	24.44	1.26*P

During the in-situ test, the control parameters include:

(1) Vehicle axle loads ranging from 0.6P to 1.2P (standard load *P* = 0.7 MPa).

(2) Traveling speeds from 5 km/h to 80 km/h.

(3) Sensor positions at 0 cm, 30 cm, 60 cm, and 160 cm below the subgrade surface.

(4) Temperature conditions at the time of testing (recorded between − 10 °C and − 25 °C for winter observations).

The evaluation indices comprise:

(1) Dynamic stress amplitude (kPa), measured under each speed and load condition.

(2) Stress distribution pattern, analyzed via isostress line profiles.

(3) Buffering depth, defined as the equivalent subgrade thickness required in the control section to match the dynamic stress attenuation observed in the test section.

These indices help quantify the mechanical performance and load-dispersing efficiency of the CRS system under real traffic conditions.

Experimental Procedure and Data Analysis:

During testing, vehicle loads were verified using axle load scales, and travel speeds were controlled using GPS-monitored speed limiters. At each load-speed combination, the vehicle passed three times over both the test and control sections to ensure repeatability. Dynamic stress data were recorded at 100 Hz using vibrating wire stress sensors connected to a synchronized data acquisition system.

**Fig. 6:**
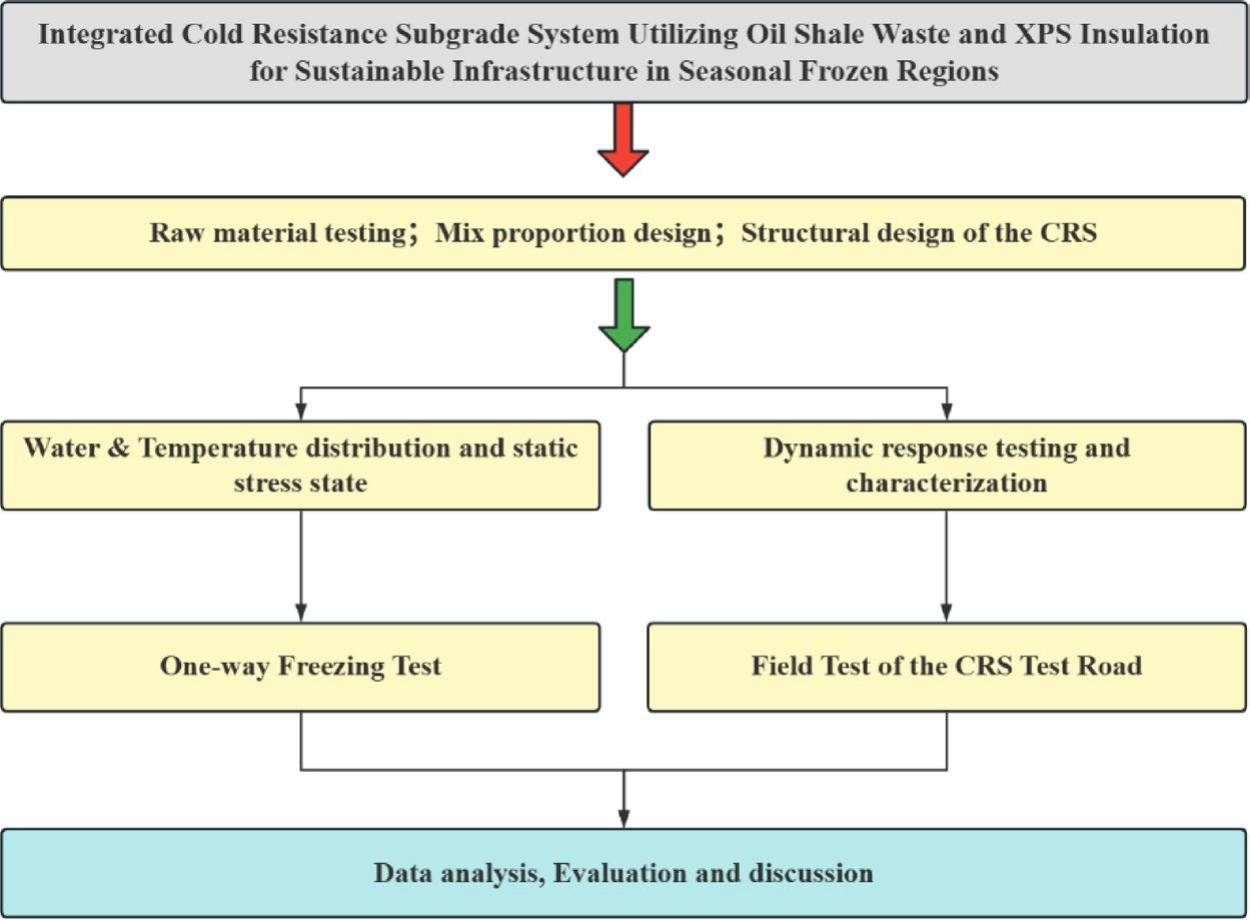
Flowchart of experimental research.

To enhance the clarity and readability of the experimental process, Fig. 6 is the experimental flowchart of this study.

## Results

### One-way freezing test

#### Temperature distribution analysis

**Fig. 7:**
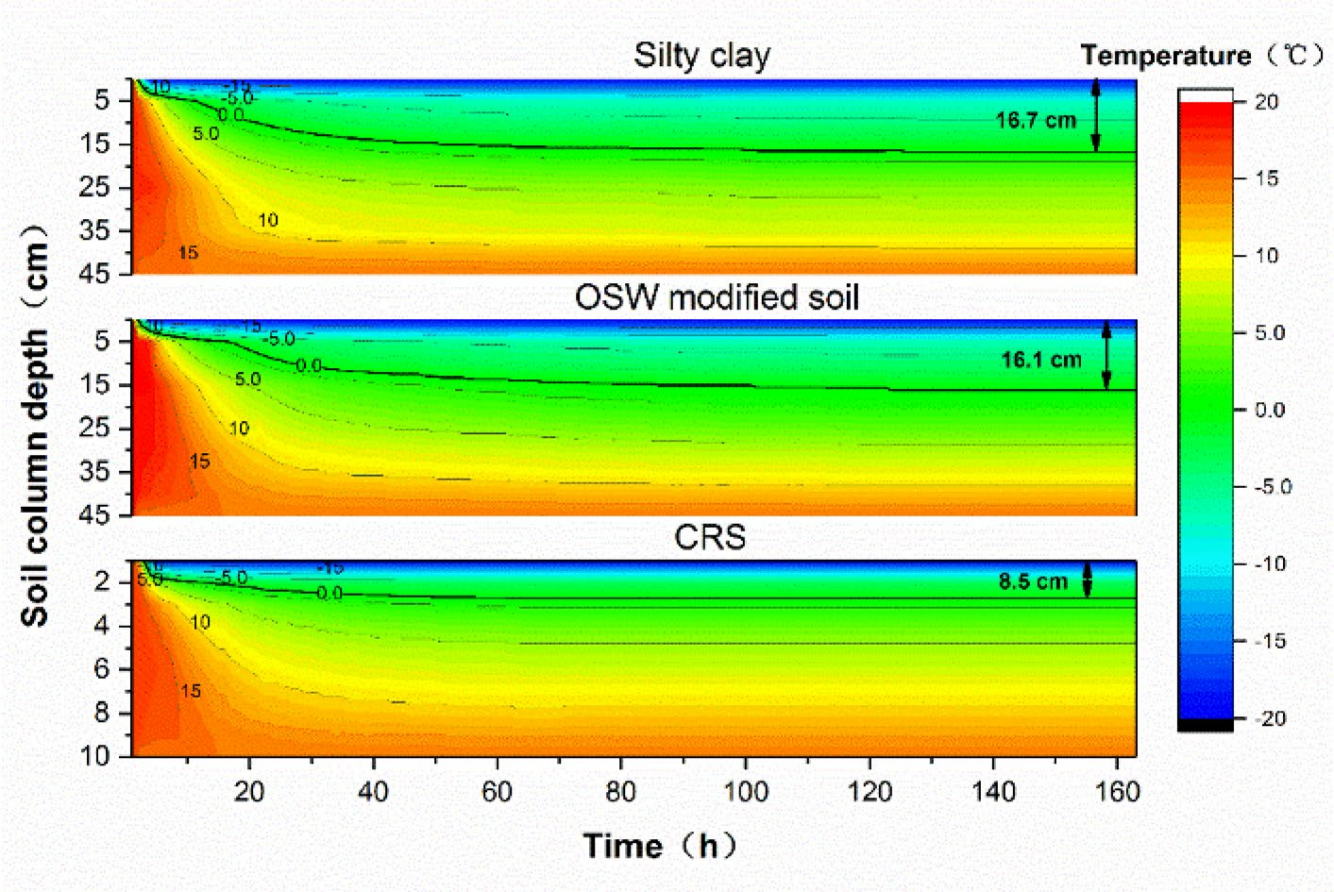
Temperature distribution diagram inside soil column.

Based on the test data, temperature distribution cloud maps were generated for the three test groups. The 0℃ isotherm was selected as the freezing line to determine its depth within the soil column under stable conditions, as shown in Fig. 7.

Under the influence of the upper and lower constant temperature tanks, the internal temperature of the soil column gradually transitions from its initial state to a stable state, where a uniform temperature gradient is established between the upper and lower sections. A comparison of the three test groups reveals that the depth of the 0℃ isotherm in the CRS decreases significantly in the stable state, reaching only 50.1% of that observed in silty clay, with a reduction in freezing depth of 8.2 cm. Similarly, the freezing depth of the CRS is 52.8% of that recorded for OSW-modified soil, with a reduction of 7.6 cm.

The reduction in freezing depth observed in the CRS group plays a critical role in enhancing frost resistance. By maintaining the 0 °C isotherm closer to the surface, the structure beneath the XPS layer remains above freezing, which effectively prevents the formation of ice lenses and associated frost heave. This thermal insulation capacity ensures that the mechanical properties of the subgrade remain stable during low-temperature periods, thereby improving the durability and service performance of the road structure in seasonally frozen regions.

#### Water content distribution analysis

**Fig. 8:**
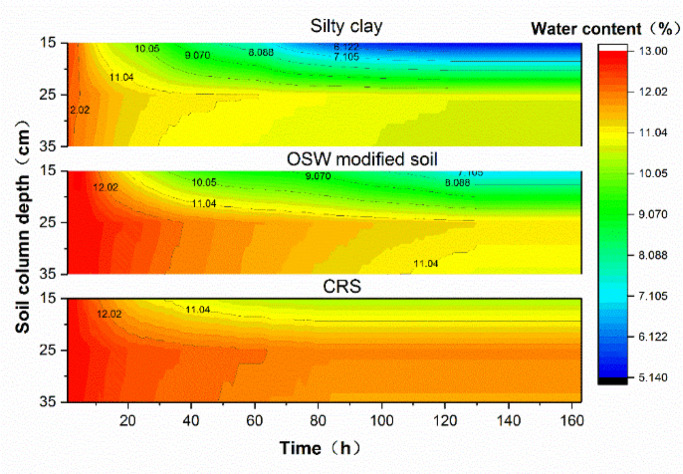
Water content distribution map inside soil column.

Based on the water content data measured at depths of 15 cm, 25 cm, and 35 cm in the soil column over the test duration, an interpolation fitting method was used to generate a cloud map of water content variations within the 15–35 cm section, as shown in Fig. 8.

As illustrated in Fig. 8, the water content within the 15–35 cm section of all three soil columns showed a decreasing trend over time under temperature loading. In this experiment, the temperature gradient was the primary factor influencing water migration. Water migration was more pronounced in the 15–25 cm section than in the 25–35 cm section, as the temperature gradient between the 15–25 cm layer and the top surface of the soil column served as the main driving force. In contrast, in the 25–35 cm section, water migration was primarily driven by the continuous positive temperature supply from the bottom of the soil column.

**Fig. 9:**
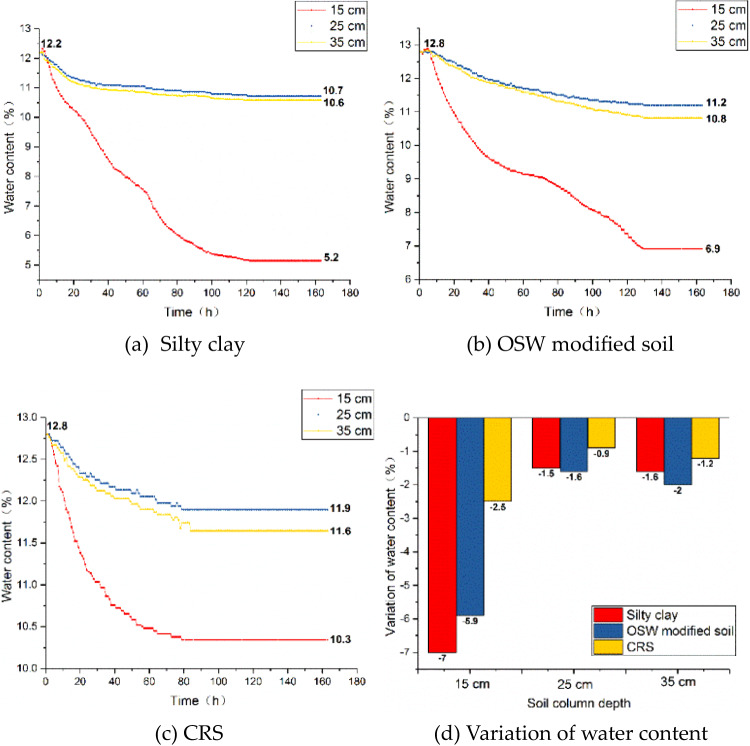
Time history curve of water content at different depth of soil column.

To further analyze the extent of water migration in the three soil groups under the same temperature gradient, time-history curves of water content at 15 cm, 25 cm, and 35 cm were plotted, as shown in Fig. 9.

Under identical temperature conditions, the silty clay group exhibited the most significant water migration, followed by the OSW-modified soil, while the CRS demonstrated the least migration. Specifically, the water migration at the 15 cm depth in the CRS was only 35.7% of that observed in the silty clay group and 42.4% of that in the OSW-modified soil group. This finding indicates that the CRS effectively mitigates the severe water migration commonly observed in subgrade soils within seasonal freezing zones under negative temperature gradients, thereby maintaining a relatively stable internal water distribution.

The significantly reduced water migration in the CRS group directly contributes to its enhanced anti-freezing performance. Excessive water movement under negative temperature gradients is a primary driver of frost heave and internal stress accumulation in subgrade materials. By maintaining a stable moisture distribution, the CRS minimizes the potential for ice segregation and volumetric expansion during freezing, effectively mitigating frost-related damage and ensuring structural integrity throughout freeze-thaw cycles.

#### Stress distribution analysis

**Fig. 10:**
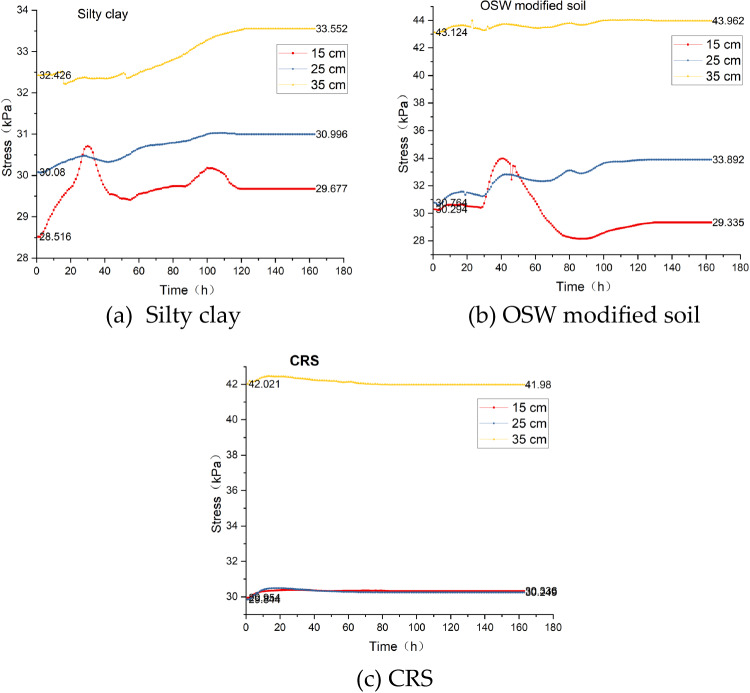
Time history curve of stress at different depth of soil column.

Figure 10 depicts the stress-time history curves of three groups of soil columns throughout the testing process. Stress variations serve as an indicator of the internal state changes within the soil.

In this experiment, the primary factors contributing to stress alterations are as follows:

1. Changes in water content induced by water migration, which is driven by the temperature gradient.​.
2. Phase transitions of water under the influence of temperature. Different states of water can alter the stress state of the soil.

As illustrated in Fig. 10, during the entire test, the stress state within the soil column of the CRS remained relatively stable. In contrast, more pronounced stress changes were observed in the soil columns of silty clay and the OSW-modified soil. The most significant stress variations occurred at a depth of 15 cm, adjacent to the upper temperature-conduction plate. Both the silty clay and OSW-modified soil columns underwent a notable process of stress increase followed by a decrease. This phenomenon is attributed to water migration and phase transitions of water under the influence of the temperature gradient. These results suggest that the CRS has the capacity to maintain the relative stability of the internal stress state of the roadbed during the freezing process.

It is noteworthy that the stress values at the 15 cm and 25 cm depths show minimal variation across all test groups. This phenomenon can be attributed to two main factors. First, both depths are located within the upper portion of the soil column where the vertical stress distribution is relatively uniform due to limited overburden pressure. Second, the presence of the XPS insulation layer in the CRS group and the homogeneous structure of the silty clay and OSW-modified soils lead to consistent mechanical response over this range, especially under static or quasi-static thermal loading conditions. Additionally, the similar temperature gradients and moisture contents at these depths result in comparable phase transitions and volumetric changes, which further contribute to the uniformity in stress response. This observation supports the structural stability and layered consistency of the tested materials.

### Field test of the CRS test road

#### Bearing capacity

The rebound deflection of the top surface represents a crucial indicator for evaluating the bearing capacity of the subgrade. The Benkelman beam method, renowned for its technological maturity and operational simplicity, is extensively employed in construction inspections to determine the rebound deflection of both subgrades and pavements.​.

**Fig. 11:**
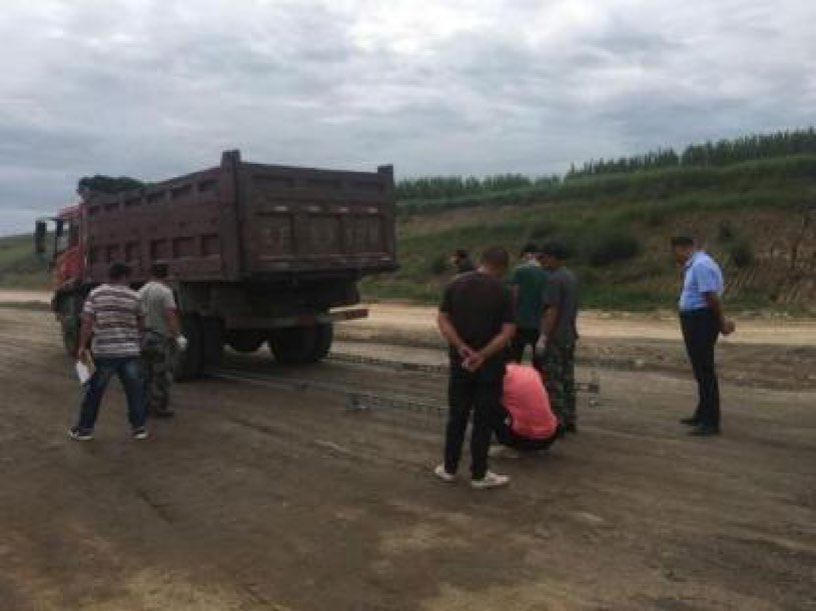
Subgrade surface deflection test.

After the construction of the OSW-modified soil layer on the test road was completed, the deflection of the subgrade top surface of the OSW - modified soil test road was detected in strict compliance with the requirements of the specification^23^, as presented in Fig. 11.

The raw data were processed following the specification requirements. The representative deflection values of the test section and the control section were 1.076 mm (mean: 82.6; standard deviation: 12.520) and 1.36 mm (mean: 90.3; standard deviation: 22.890), respectively. Both values met the design requirement of being less than 1.791 mm. By comparing the representative values, mean values, and standard deviations of the deflection, it was evident that the deflection value of the test section was lower than that of the control section, with relatively lower discreteness. This indicates that the subgrade of the test section exhibits higher bearing capacity, greater uniformity, and enhanced stability.

There exists a well-established relationship between the deflection and the resilient modulus of the subgrade. In accordance with the calculation formula (Formula 1) provided in the specification^20^, the resilient modulus of the subgrade was back-calculated.

1 $$\:l={9308E}^{-0.938}$$

Where E denotes the resilient modulus of subgrade, $\:l$ is the rebound deflection of the subgrade.

The design documents indicate that the test road has a heavy-traffic load grade. According to the design specifications for asphalt pavements, the resilient modulus of the subgrade should be greater than or equal to 50 MPa. The resilient moduli of the test section and the control section, calculated using Formula 1, were 65.61 MPa and 52.67 MPa, respectively. These values meet the specification requirements, demonstrating that the bearing capacity of the OSW-modified soil used as a highway subgrade complies with the relevant specifications.

 Compared with traditional sand and gravel subgrades commonly used in cold regions, the CRS demonstrates superior bearing capacity and uniformity. According to the existing research results^24,25^, typical resilient modulus values for cement-treated aggregate base range from 40 to 60 MPa, whereas the CRS test section achieved 65.61 MPa. Moreover, the representative rebound deflection of the CRS Sect. (1.076 mm) is significantly lower than that of conventional structures, indicating improved load dispersion and long-term stability.

#### Cold resistance performance

**Fig. 12:**
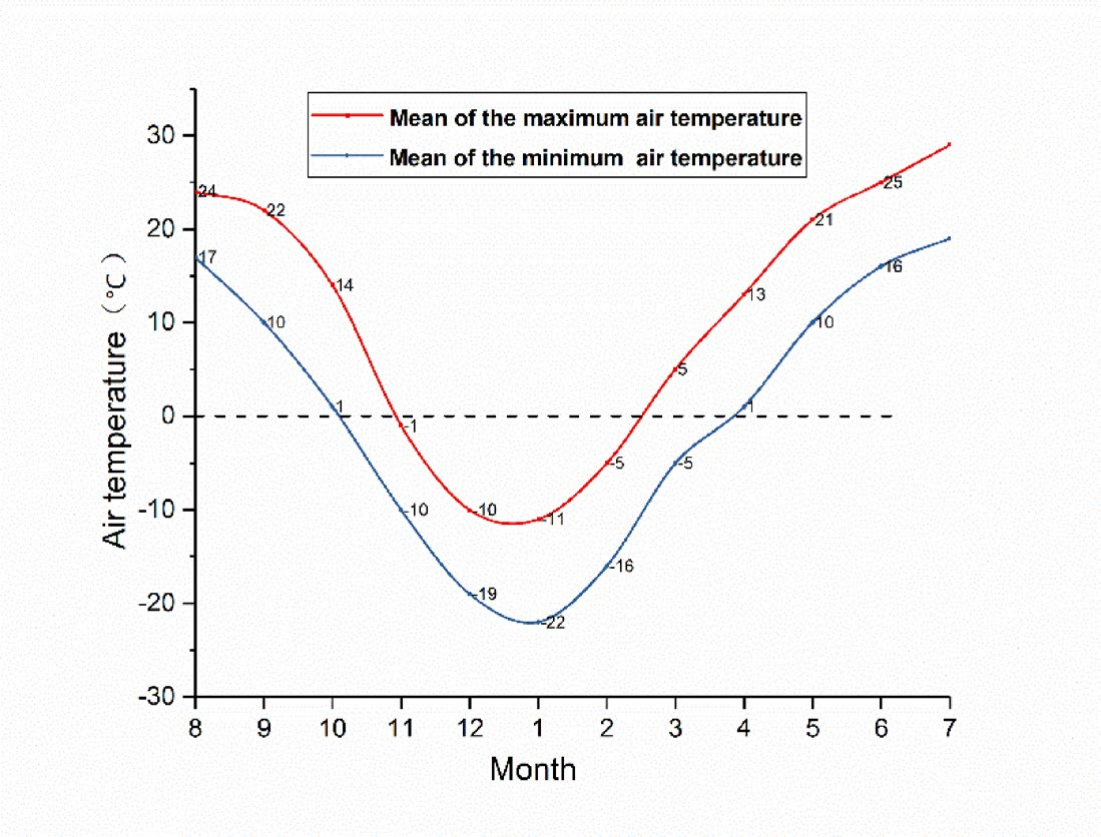
Monthly mean temperature map of the location of the test road.

**Fig. 13:**
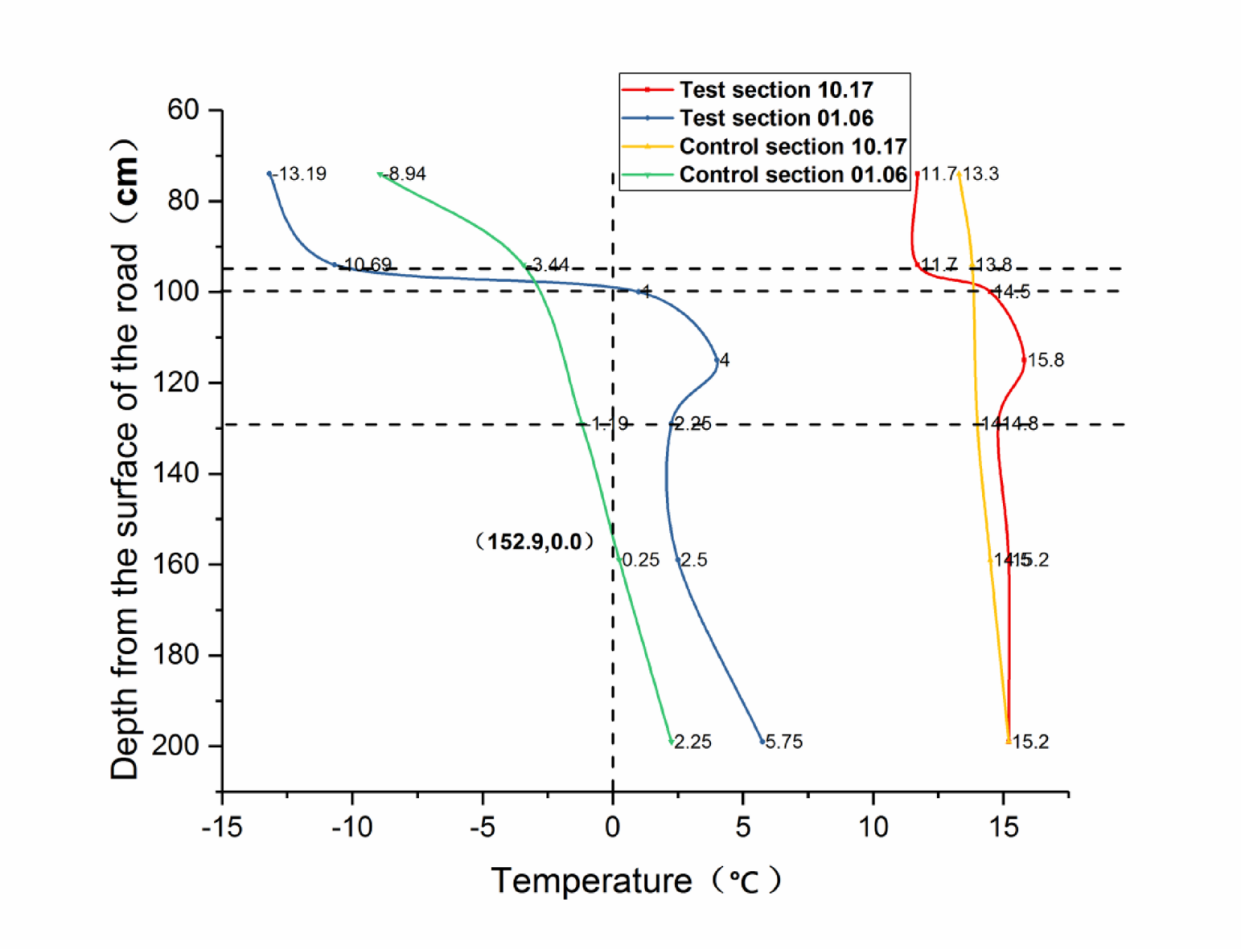
Temperature distribution of two sections.

The local monthly average temperatures are presented in Fig. 12. The data source is http://www.tianqi.com/qiwen/city_yushu/. Temperature data were collected on October 17 and January 6 of the subsequent year. Figure 13 illustrates the distribution of temperature data for these two time segments.

When examining Figs. 12 and 13 in tandem, it becomes evident that the first temperature data collection occurred during the early freezing stage. At this time, the atmospheric temperature experienced a significant decline, while the internal temperature of the road structure remained relatively high. As a consequence, energy exchange between the atmosphere and the road structure led to a progressive reduction in the internal temperature of the road structure. Owing to the insulating property of the XPS plate within the CRS, energy at the bottom of the XPS plate could not be exchanged with the structure above the plate. As a result, the temperature above the XPS plate was 2 °C lower than that at the same depth within the sand-and-gravel subgrade. Conversely, the temperature below the XPS plate was marginally higher than that of the sand-and-gravel subgrade.

The second temperature data collection took place during the month with the lowest annual temperature. The air temperature at the time of data collection was − 30 °C. During this month, the road structure was most severely affected by the negative-temperature energy of the atmosphere, and the road structure gradually reached its maximum freezing depth. By comparing the temperature data of the test section and the control section, it is clear that the freezing depth of the control section reached 152.9 cm, whereas that of the test section was only 94 cm. The freezing line remained at the top of the XPS plate. The temperature difference between the upper and lower surfaces of the plate reached 11.69 °C, and the temperature below the plate remained above 0 °C. The subgrade soil beneath the plate did not freeze. This phenomenon demonstrates the outstanding cold-resistance properties of the CRS.

#### Dynamic stress response

**Fig. 14:**
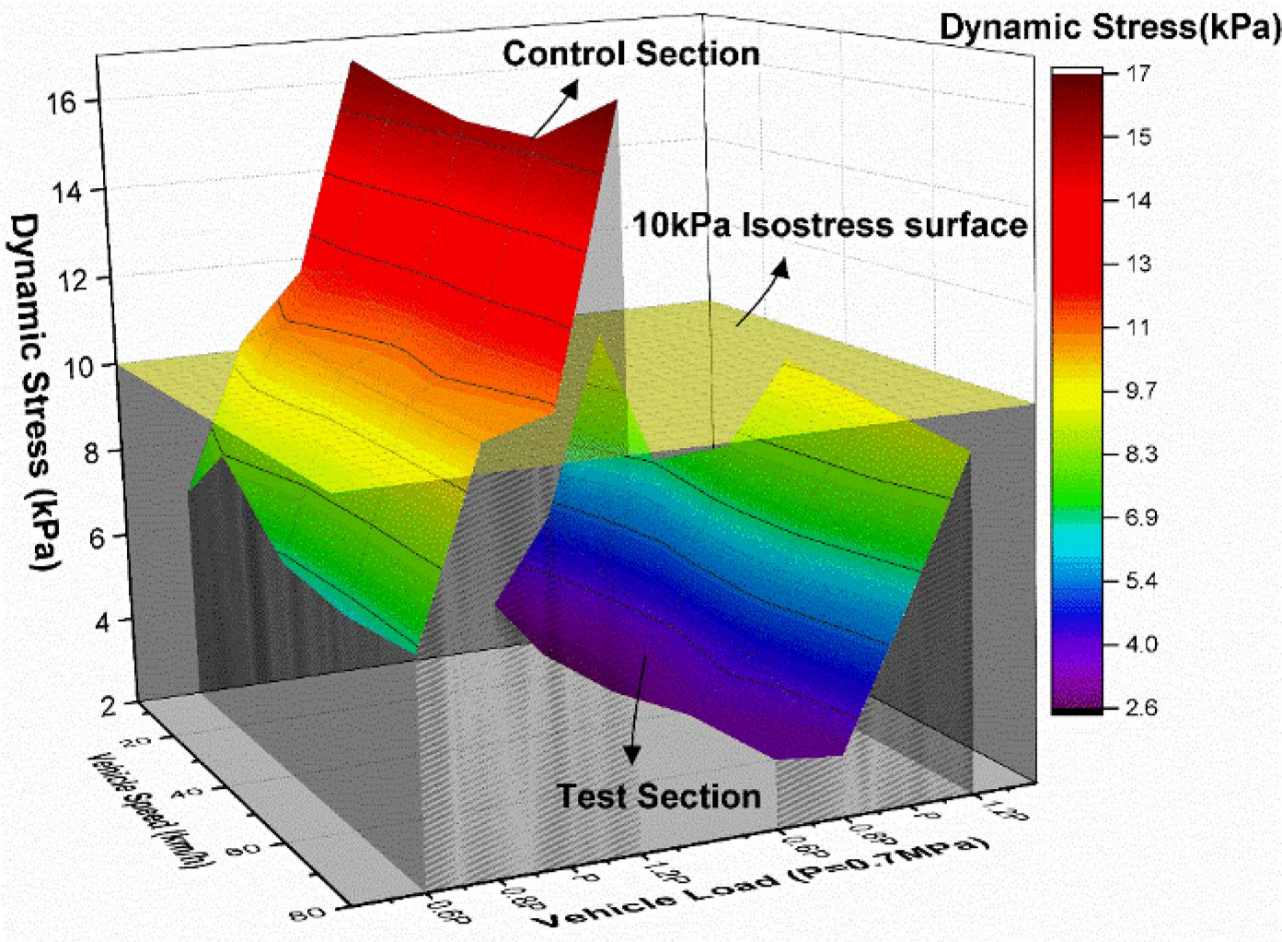
The dynamic stress response surface diagram of the position І of the two sections under different loading conditions.

To investigate the dynamic stress response characteristics of the Cold Resistance Structure (CRS), dynamic stress distributions were analyzed at three depths beneath the XPS plate in the test section: 0 cm (Position І), 30 cm (Position II), and 60 cm (Position III). Comparative assessments were conducted with equivalent depths in the control section. Figure 14 illustrates the dynamic stress response profiles of both sections under varying load conditions.

Significant differences in dynamic stress responses are observed between the two sections. The test section exhibits consistently lower dynamic stress magnitudes and reduced response surface areas compared to the control section. This indicates that the CRS experiences smaller dynamic stresses under identical load conditions and demonstrates diminished sensitivity to load variations, thereby enhancing structural stability.

**Fig. 15:**
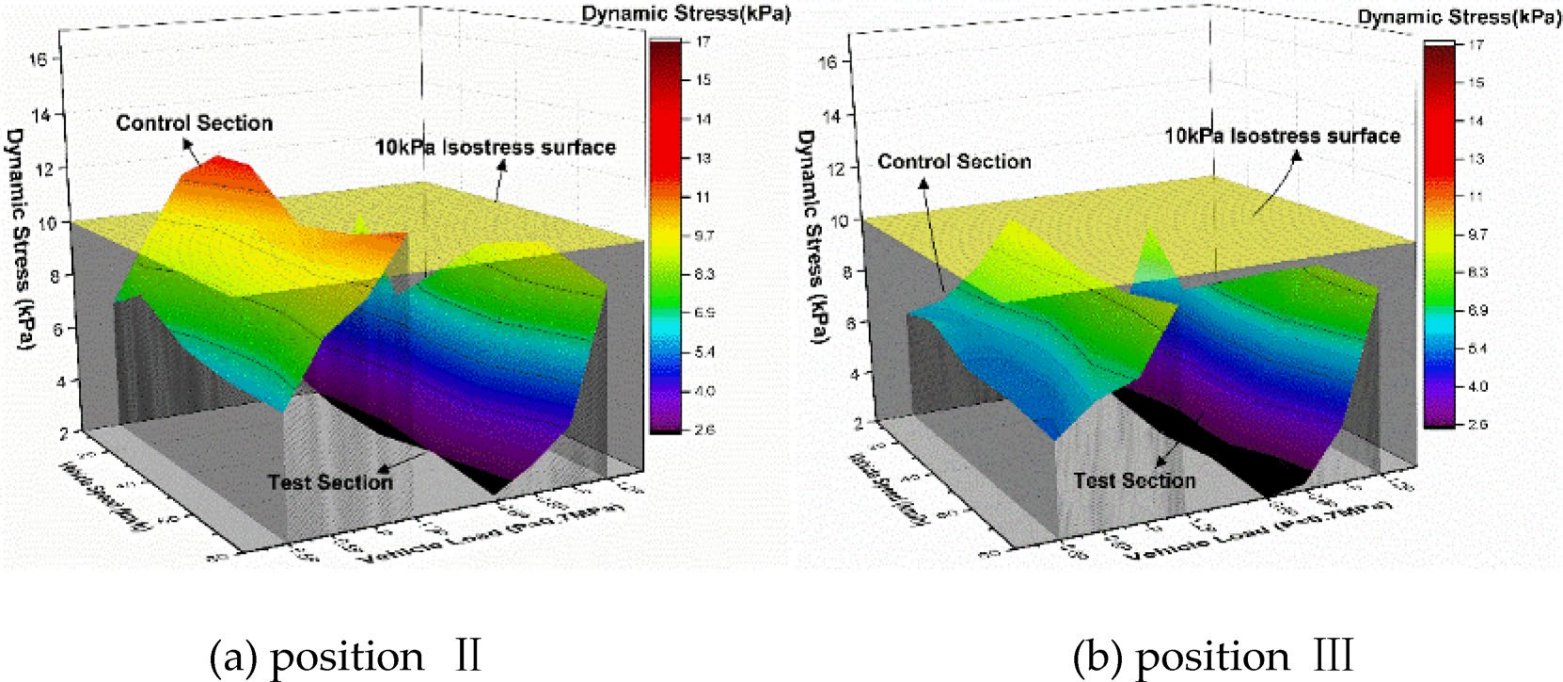
The dynamic stress response surface diagram of the position Ⅱ and Ⅲ of the two sections under different loading conditions.

Figure 15 further details dynamic stress responses at Positions II and III. Across all measurement depths, the test section maintains lower stress levels than the control section. Stress magnitudes diminish with increasing structural depth in both sections, though the control section exhibits pronounced stress gradients (7–20 kPa range) with dense isostress line distributions. These abrupt stress transitions between adjacent structural layers contrast with the test section’s uniform stress distribution (predominantly < 5 kPa) and wider isostress line spacing, reflecting superior stress dispersion characteristics.

**Fig. 16:**
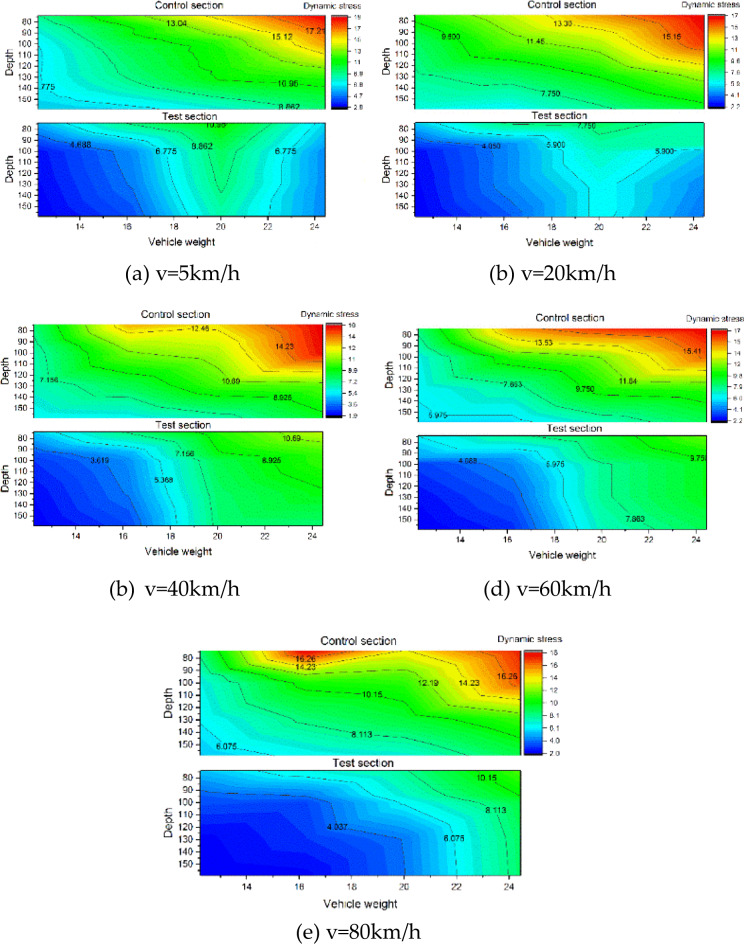
Cloud diagram of dynamic stress response of subgrade structure under different vehicle weights.

**Fig. 17:**
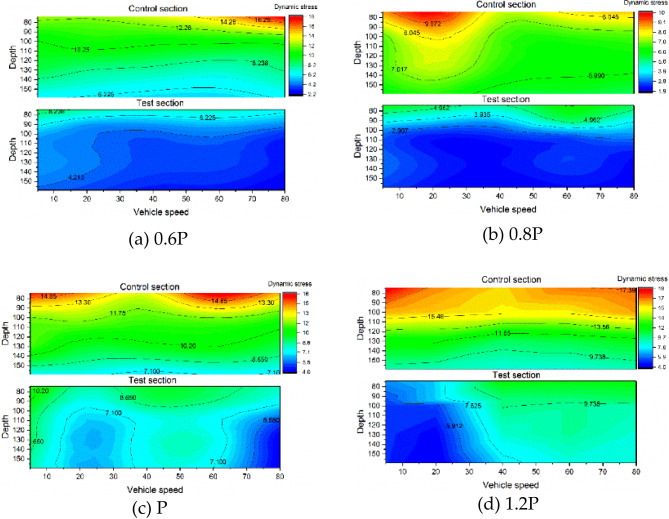
Cloud diagram of dynamic stress response of subgrade structure under different vehicle speeds.

To systematically evaluate vehicle parameter effects, stress distributions under varying speeds and loads are presented in Figs. 16 and 17. Figure 16 demonstrates load-dependent stress escalation in both sections. The control section displays concentrated stress gradients (7–20 kPa) with steep interlayer transitions, while the test section maintains a uniform 2–10 kPa range. This stress homogenization arises from the XPS plate’s honeycomb structure, which absorbs and decelerates load waves, enabling gradual stress propagation through underlying layers.

Figure 17 reveals negligible correlations between vehicle speed and dynamic stress magnitudes across both sections. This aligns with established findings that typical highway speeds fall outside structural resonance frequencies^26^, confirming the CRS’s compatibility with existing road dynamics.

**Fig. 18:**
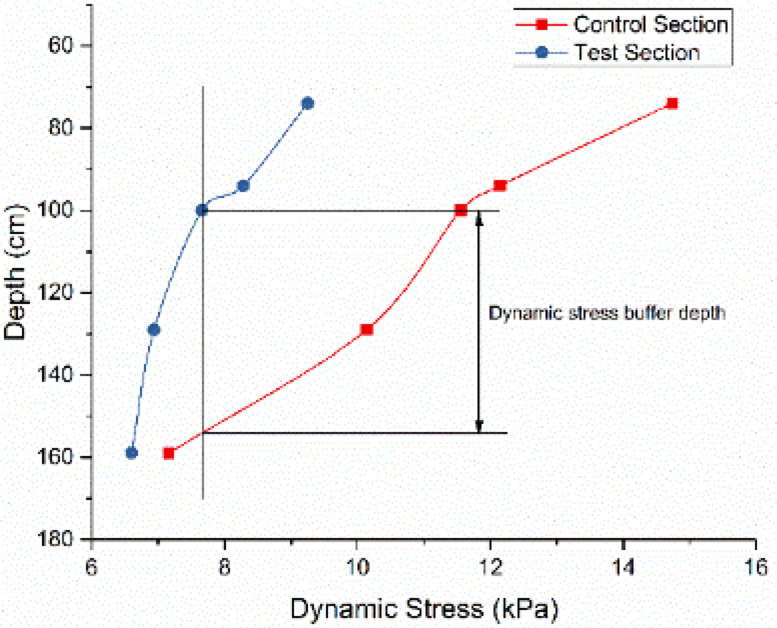
Schematic diagram of effective buffer depth of dynamic stress.

**Fig. 19:**
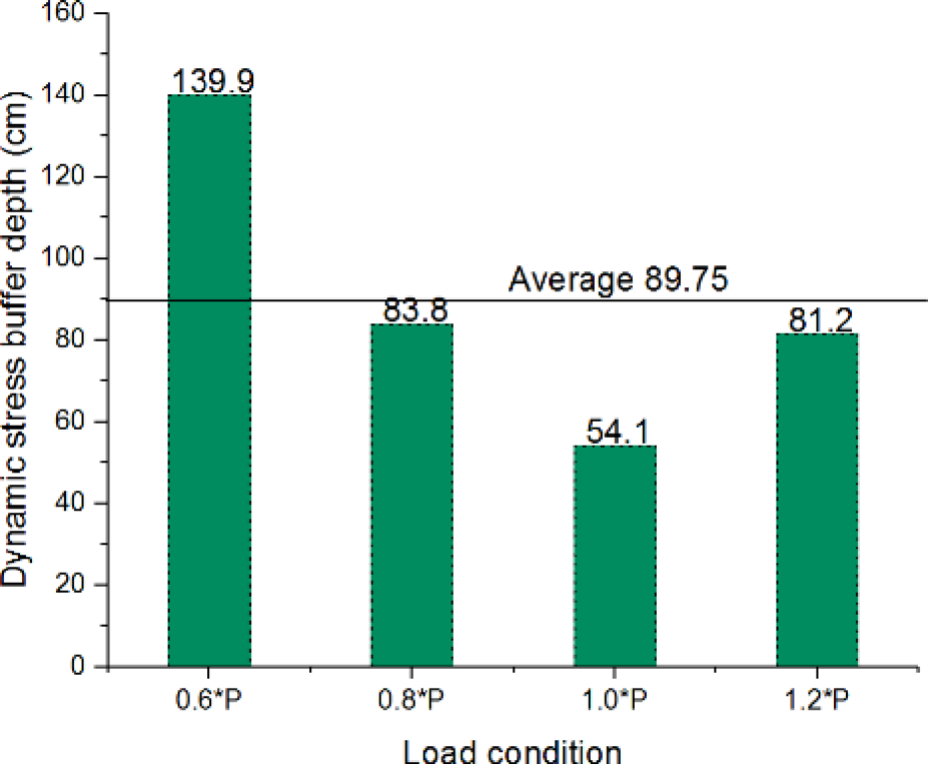
Schematic diagram of effective buffer depth of dynamic stress under different loading conditions.

Notably, dynamic stresses at the control section’s maximum depth (160 cm) approximate those at the test section’s shallow depth (75 cm). This 85 cm differential quantifies the XPS plate’s effective buffering capacity. As vehicle speed variations showed no systematic stress influence, dynamic stresses were averaged across speeds for equivalent load conditions. Figure 18 illustrates the buffering depth determination methodology for load condition P, while Fig. 19 summarizes results across four load scenarios.

The analysis identifies an average equivalent buffering depth of 89.75 cm for the XPS plate, corresponding to the performance of conventional gravel subgrade thickening. This stress attenuation mechanism significantly reduces traffic load disturbances, enhancing subgrade durability and service life.

## Discussions

### Environmental evaluations

To assess potential environmental impacts of OSW-modified soil subgrade, leaching solution toxicity tests were conducted and evaluated against China’s Standard for Groundwater Quality^27^, as detailed in Table 6. All measured parameters comply with regulatory thresholds, confirming the material’s environmental compatibility and minimal risk to adjacent groundwater systems.

**Table 6:** Environmental assessment data of OSW modified soil leaching solution.

Anion and cation	Na^+^	Ca^2+^	Mg^2+^	NH_4_^+^	Cl^−^	NO_3_^−^	F^−^
Leachate (mg/L)	7.05	14.64	2.64	0.08	6.97	3.44	0.73
Standard value (mg/L)	100	150	-	0.5	250	20	1.0
Evaluation	√	√	-	√	√	√	√

Element	Cu	Pb	Co	Zn	Cd	Ni	Al
Leachate (mg/L)	0.0044	0.0011	0.0003	0.0409	0.0003	0.0030	0.1030
Standard value (mg/L)	1.0000	0.0100	0.0500	1.0000	0.0050	0.0200	0.2000
Evaluation	√	√	√	√	√	√	√

Element	Cr	Hg	Ag	TFe	Sb	As	Ba
Leachate (mg/L)	0.0134	0.0001	0.0003	0.0030	0.0003	0.0071	0.0750
Standard value (mg/L)	0.0500	0.0010	0.0500	0.3000	0.0050	0.0100	0.7000
Evaluation	√	√	√	√	√	√	√

Although the short-term leaching test results indicate that the OSW-modified soil complies with environmental safety standards, it is worth noting that subgrade materials may undergo gradual chemical transformations under prolonged environmental exposure, particularly under alternating dry-wet and freeze-thaw cycles. These conditions may potentially enhance the mobility of certain heavy metals or increase the leaching concentration over time. Therefore, the long-term leaching behavior and cumulative environmental impacts of OSW-modified subgrade materials warrant further investigation through accelerated leaching simulations or field monitoring. Incorporating long-term environmental performance assessments into future studies will help ensure the ecological safety and regulatory compliance of CRS applications throughout their full life cycle.

### Material cost comparison

Economic analysis compares CRS and conventional subgrade structures using actual engineering data (Table 7). According to the design document of the highway where the test road is located, the conventional subgrade structure is replaced with 1.2 m sand and gravel below the top of the subgrade, and the width is 31 m.

**Table 7:** Subgrade material cost table(/km).

Section	Material	Volume (m^3^)	Price ($/m^3^)	Material cost ($)	Total ($)
Control Section	Sand and Gravel	37,200	16.83	626,076	626,076
Test Section	XPS	1550	73.88	114,508	356,166
	Oil Shale Waste	2633	84.43	222,305	
	Fly Ash	1610	12.02	19,353	

As can be seen from Table 7, the material cost of the CRS has been reduced by $ 269,910/km compared with the conventional structure, and the material cost has been reduced by about 43%, which is a very considerable figure. The cost of OSW in the table is mainly the transportation cost, and the waste residue itself is free of charge. In promotional applications, the cost savings are about us $ 222,305/km if the surrounding materials are available.

In addition, according to Environmental Protection Tax Law of the People’s Republic of China, OSW and fly ash belong to solid waste residue, whose environmental disposal fee is $ 3.535/ton every year. The CRS can consume a total of 9318.6 tons of OSW and fly ash per kilometer, and the cumulative cost of environmental protection disposal can reach $ 32,942/year.

### Application promotion and long-term monitoring

Adaptation strategies for varying highway grades require proportion adjustments through laboratory testing. Table 8 presents California Bearing Ratio (CBR) values for five modified soil mixtures, all meeting specification requirements for highway applications.

**Fig. 20:**
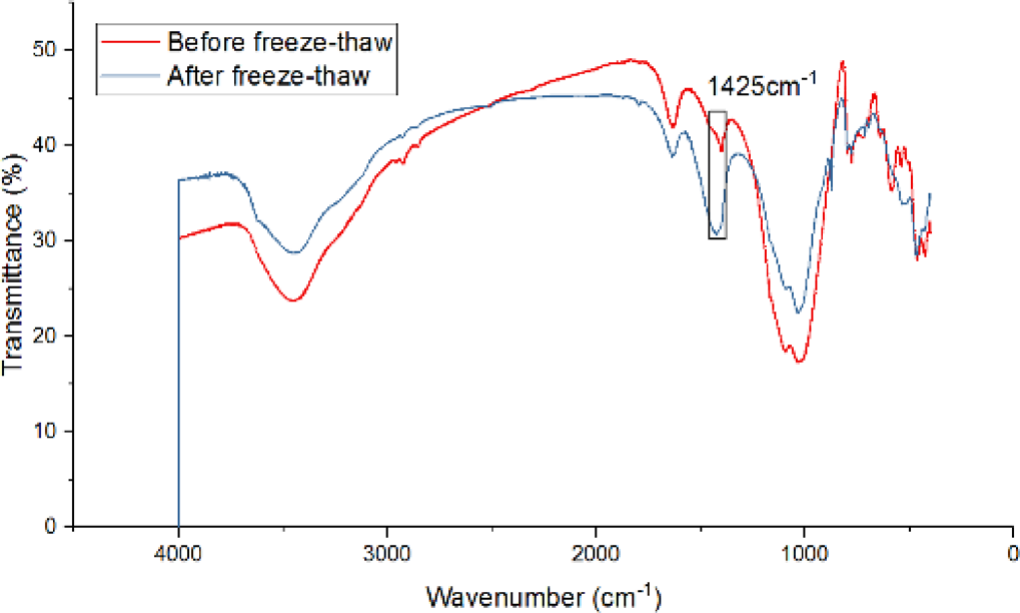
Fourier transform infrared spectrum analysis of OSW modified soil before and after freezing and thawing^21^.

Fourier-transform infrared spectroscopy analysis reveals intensified carbonate vibration peaks (1425 cm⁻¹) after five freeze-thaw cycles (Fig. 20), indicating calcium carbonate formation that enhances material strength. These findings necessitate long-term performance monitoring of the test road, with subsequent studies planned to evaluate durability under extended service conditions.

**Table 8:** **CBR values of** OSW **modified soil with different mix ratios**.

Ratio of dry mass	CBR (%)	Specification requirements
OSW: Fly Ash: Silty Clay = 3: 4: 3	13.4	≥ 8 (Highway)
OSW: Fly Ash: Silty Clay = 4: 3: 3	17.5	
OSW: Fly Ash: Silty Clay = 7: 5: 8	39.1	
OSW: Fly Ash: Silty Clay = 9: 3: 8	41.4	
OSW: Fly Ash: Silty Clay = 2: 1: 2	44.9	

## Conclusions

This study proposes a novel Cold Resistance Structure (CRS) that integrates extruded polystyrene (XPS) insulation with oil shale waste (OSW)-modified subgrade soil, targeting the frost resistance challenges in seasonal frozen regions. Through laboratory tests and full-scale field validation, the following conclusions are drawn:

(1) Enhanced frost protection: The CRS system reduces subgrade freezing depth by up to 52.8%, maintains a positive temperature below the XPS plate during extreme cold, and elevates the frost line above critical structural layers.

(2) Moisture and stress stability: Compared with silty clay, the CRS reduces water migration by 64.3% at shallow depths and effectively suppresses internal stress fluctuations during freezing due to thermal buffering.

(3) Superior mechanical performance: Field tests show a 21% lower subgrade deflection and 25% higher resilient modulus in the test section versus the control section, ensuring better load-bearing capacity and long-term stability.

(4) Outstanding dynamic stress attenuation: The CRS achieves an equivalent stress buffering depth of 89.75 cm, substantially mitigating the impact of vehicle loads and extending subgrade service life.

(5) Economic and environmental benefits: The CRS reduces material costs by 43% per kilometer, consumes over 9300 tons of OSW and fly ash, and avoids annual disposal fees of approximately $33,000, demonstrating a scalable green solution for cold-region infrastructure.

This integrated design offers a novel and sustainable subgrade system, combining energy-efficient insulation and large-scale industrial waste reuse, with strong applicability in cold region engineering and circular economy practices.

## Data Availability

All data supporting the results of this study are available in the article. They can also be obtained from the corresponding author upon reasonable request.
